# Association between ambient PM_2.5_ exposure during pregnancy and risk of preterm birth: a retrospective birth cohort study in Chengdu, Southwest China

**DOI:** 10.3389/fpubh.2026.1936547

**Published:** 2026-09-09

**Authors:** Yi Zhang, Keyao Lv, Yuping Yuan, Ruifeng Li, Xinbin Li

**Affiliations:** 1 Wuhou District People’s Hospital/Wuhou District Health Hospital for Women & Children, Chengdu, Sichuan, China; 2 Department of Occupational and Radiological Prevention and Control, Mianyang Center for Disease Control and Prevention, Mianyang, Sichuan, China

**Keywords:** ground monitoring, PM2.5, population heterogeneity, preterm birth (PTB), satellite-based exposure

## Abstract

**Background:**

Prenatal PM_2.5_ exposure has been associated with preterm birth (PTB), but evidence on exposure windows and population heterogeneity remain inconsistent.

**Methods:**

This retrospective cohort included 35,136 singleton births at a Chengdu hospital from 2015 to 2024. Satellite- and monitor-based PM_2.5_ exposures were estimated for conventional and fixed gestational windows. Logistic regression adjusted for individual characteristics, meteorological factors, conception season, and district fixed effects, with cluster-robust standard errors by district and last-menstrual-period year-month. PTB was classified as earlier (28–33 weeks) or late (34–36 weeks), and exploratory subgroup analyses were conducted.

**Results:**

Among 35,136 births, 2,622 (7.46%) were preterm. Mean whole-pregnancy PM_2.5_ concentrations were 49.11 *ug/m^3^* for satellite data and 51.92 *ug/m^3^* for monitoring data. Per 10 *ug/m^3^* increase, the whole-pregnancy ORs were 1.089 (95% CI: 1.010–1.174) and 1.091 (95% CI: 0.996–1.195), respectively. Positive associations were observed during the first and third trimesters. For exposure during weeks 32–33, the ORs for late PTB were 1.063 and 1.069 using monitoring and satellite data, respectively; corresponding ORs for exposure during weeks 34–35 and PTB at week 36 were 1.084 and 1.095. Positive estimates occurred for late but not earlier PTB. Exploratory analyses suggested potential heterogeneity by ethnicity, parity, and migrant status.

**Conclusion:**

Prenatal PM_2.5_ exposure was associated with PTB,with positive associations observed for the first and third trimesters and in the landmark analyses of weeks 32–33 and 34–35. Satellite and monitoring data yielded similar district-level estimates, while satellite data provided complete coverage and narrower confidence intervals. Reducing PM_2.5_ exposure throughout pregnancy may help reduce PTB burden.

## 1 Introduction

Preterm birth (PTB), defined as delivery before 37 completed weeks of gestation, remains one of the leading causes of neonatal mortality and long-term morbidity worldwide (1). Approximately 15 million infants are born preterm each year, accounting for nearly 1 million deaths from PTB-related complications (2). Survivors are at increased risk of respiratory diseases, neurodevelopmental disorders, cognitive impairment, and other adverse health outcomes that persist throughout life (3).

Maternal exposure to ambient fine particulate matter with an aerodynamic diameter ≤ 2.5 um (PM_2.5_) has been associated with an increased risk of PTB in different populations (4–6). However, the magnitude of the association and the gestational period showing the strongest association have varied across studies. A meta-analysis by Sun et al. reported a pooled odds ratio of 1.13 (95% CI: 1.03–1.24) for each 10 *ug/m^3^* increase in whole-pregnancy PM_2.5_ exposure (7). In a national cohort of 1,280,524 singleton pregnancies using satellite-based estimates, Li et al. (8) reported stronger associations during the first and second trimesters and the entire pregnancy than during the third trimester. By contrast, Guo et al. (9) analyzed 426,246 pregnancies using ground-monitoring data and found the strongest association during the third trimester (HR = 1.06, 95% CI: 1.06–1.07 per 10 *ug/m^3^* increase). Chu et al. (10) reported associations for first-trimester and whole-pregnancy exposure in a multicentre Chinese cohort using satellite-based estimates, whereas Zhou et al. (11) found stronger associations during late pregnancy among 572,116 births in Chongqing using monitoring data. Guo et al. (9) further summarized the available Chinese evidence and reported increased risks of PTB associated with PM_2.5_ exposure during the second trimester, third trimester, and entire pregnancy, whereas no significant association was found for first-trimester exposure. Therefore, additional regional studies using clearly defined gestational windows are needed to assess the consistency of gestational-window-specific associations across populations.

Ground-monitoring measurements and satellite-based estimates are commonly used to assess ambient PM_2.5_ exposure, but differences in their spatial coverage, resolution, and exposure assignment may contribute to variation in effect estimates. Hyder et al. (12) compared monitor-based and satellite-based estimates in a birth cohort from Connecticut and Massachusetts. The estimated associations differed for birth weight and small for gestational age, whereas none of the exposure approaches showed a significant association with PTB. In a Shanghai birth cohort, Xiao et al. (13) compared gap-filled satellite estimates, satellite estimates without gap filling, and central-site measurements. Estimates based on gap-filled satellite data were stronger than those based on satellite data without gap filling and were similar in magnitude to those based on central-site measurements, with narrower confidence intervals. These studies suggest that the influence of exposure data may differ across birth outcomes and study settings. In addition, several large Chinese cohort studies reported differences in the association between prenatal PM_2.5_ exposure and PTB by maternal age, reproductive history, residential status, and infant gender (8–10). Therefore, additional regional studies using different exposure datasets may help determine whether estimated associations differ by exposure-assessment method and population characteristics.

Chengdu, located in the Sichuan Basin in Southwest China, has experienced relatively high levels of PM_2.5_ pollution. Its basin topography and meteorological conditions can restrict pollutant dispersion, while rapid urbanization, dense traffic, and industrial emissions contribute to local PM_2.5_ concentrations. As a major regional medical center, Chengdu serves patients from both local and migrant populations, providing a suitable setting for examining differences in PM_2.5_-associated PTB risk across population subgroups.

In this hospital-based retrospective birth cohort of 35,136 singleton births in Chengdu, we used satellite- and monitor-based PM_2.5_ data to: (1) estimate the associations of PM_2.5_ exposure during conventional and fixed gestational windows with PTB; (2) examine the associations with earlier and late PTB; and (3) explore differences by maternal age, ethnicity, parity, migrant status, and infant gender.

## 2 Methods

### 2.1 Study region

**Figure 1:**
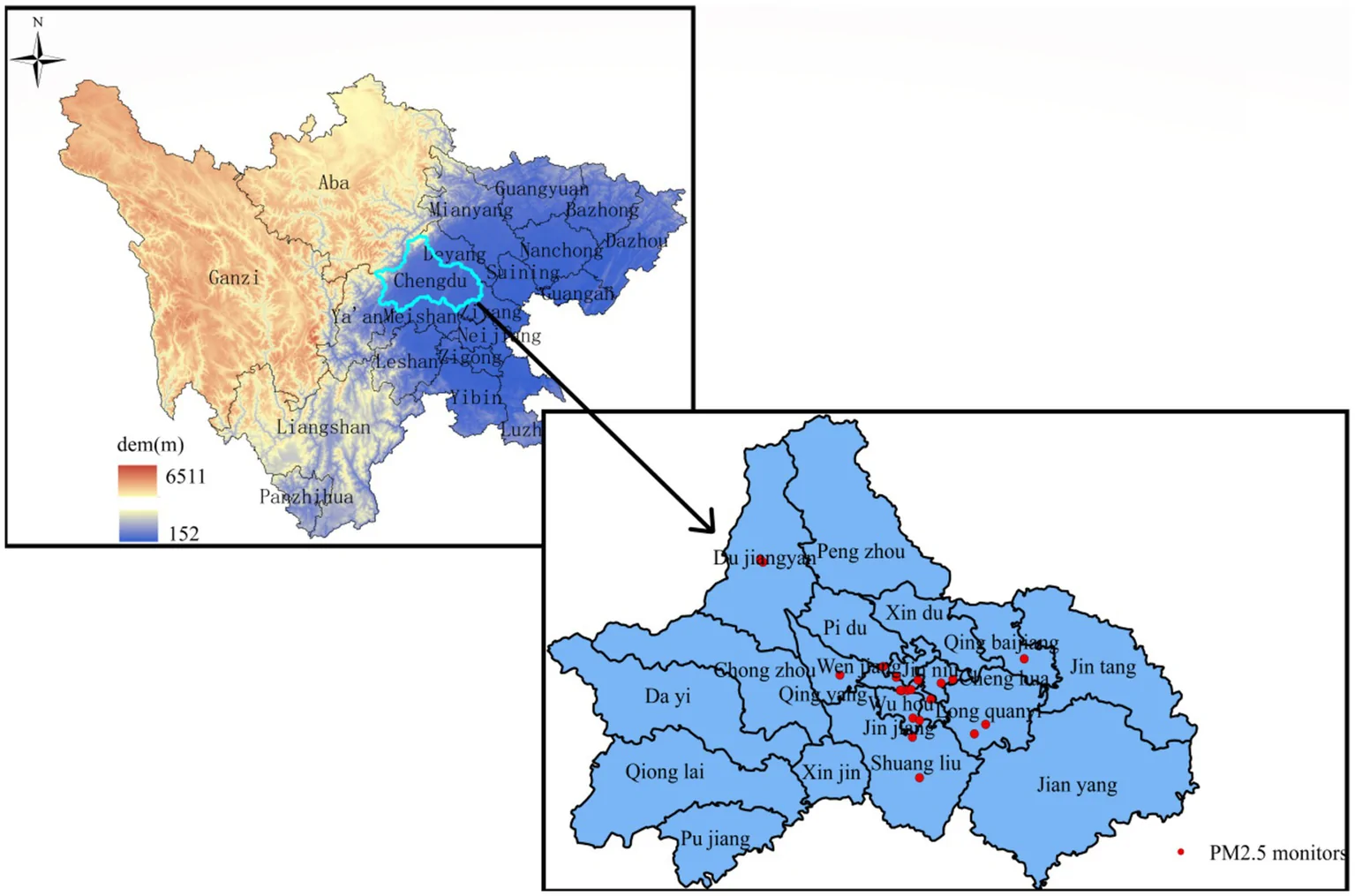
The map of 20 districts and 23 PM_2.5_ monitors distribution in Chengdu.

Chengdu (102°54′E-104°53′E, 30°05′N-31°26′N) covers an area of 14,335 km^2^ and includes 20 districts (Figure 1). As a major city in Southwest China, Chengdu is located in the western part of the Sichuan Basin, one of the four major pollution zones. The city has a permanent population of over 20 million and more than 6 million registered motor vehicles, ranking second nationwide. As the provincial capital of Sichuan Province and a major gateway linking the Sichuan Basin with Tibetan areas in western Sichuan, Chengdu has long attracted substantial inflows of ethnic minorities, particularly Tibetans, due to its locational advantages in trade, education, healthcare, and religious activities (14).

### 2.2 Data collection and processing

#### 2.2.1 Delivery data

The delivery data were collected from Wuhou District Women and Children’s Health Hospital between January 1, 2015, and December 31, 2024. This hospital-based retrospective birth cohort included all eligible singleton live births recorded during the study period. The hospital database contained information on gestational age at delivery, delivery date, maternal district of residence, ethnicity, and other available maternal and neonatal characteristics. Gestational age was determined from the recorded LMP for 34,389 births (97.87%). For 747 births (2.13%) without a recorded LMP, the LMP date was back-calculated from the clinically recorded gestational age and delivery date. The first trimester was defined as gestational weeks 1–13, the second trimester as weeks 14–27, and the third trimester as week 28 to delivery. We additionally calculated exposure during four fixed windows: weeks 0–27, 28–31, 32–33, and 34–35 weeks. The weeks 28–31 analysis was restricted to pregnancies ongoing at week 32 and evaluated subsequent PTB occurring during weeks 32–36. The weeks 32–33 analysis was restricted to pregnancies ongoing at week 34 and evaluated subsequent PTB occurring during weeks 34–36. The weeks 34–35 analysis was restricted to pregnancies ongoing at week 36 and evaluated PTB occurring during week 36. Each landmark model estimated a conditional association among pregnancies that remained ongoing through the corresponding exposure window. These landmark estimates represent conditional associations in different risk sets and should not be directly compared across gestational windows.

#### 2.2.2 PM_2.5_ data

Daily satellite-based PM_2.5_ data were obtained from the ChinaHighPM_2.5_ dataset^1^ from 2014 to 2024. The ChinaHighPM_2.5_ dataset performed a cross-validation coefficient of determination (CV-R^2^) of 0.92, a root-mean-square error (RMSE) of 10.76 *ug/m^3^*, and a mean absolute error (MAE) of 6.32 *ug/m^3^*.

The monitor-based PM_2.5_ concentrations from 2014 to 2024 were obtained from the China Environmental Monitoring Center (CNEMC).^2^ Monitor-based PM_2.5_ data were obtained from 23 stations in 11 of the 20 study districts. Measurements from stations within the same district were averaged by day. Missing district-day concentrations were estimated using inverse-distance weighting based on the three nearest valid stations, followed by temporal interpolation or endpoint extension when necessary. Analyses restricted to pregnancies with original monitoring observations were also estimated, and detailed information are provided in Supplementary Table S1.

For each pregnancy, daily district-level PM_2.5_ concentrations were averaged over the first trimester (weeks 1–13), second trimester (weeks 14–27), third trimester (week 28 to delivery), and entire pregnancy (LMP to delivery). For sensitivity analyses addressing differences in exposure-window duration, we additionally calculated PM_2.5_ exposure during two fixed gestational windows: weeks 0–27 and weeks 28–31.

#### 2.2.3 Meteorological data

Daily 2-m air temperature (t2m), 10 m u-component of wind (u10), 10 m v-component of wind (v10) and total precipitation (tp) data were extracted from the European Center for Medium-Range Weather Forecasts (ECMWF)^3^ with a resolution of 0.125^°^ × 0.125^°^. The wind speed was derived according to u10 and v10, and the temperature was calculated from the t2m (15). Then, we calculated the average meteorological data at the district level.

### 2.3 Statistical analysis

Covariates were specified *a priori* based on previous evidence, clinical relevance, and their plausible relationships with PM_2.5_ exposure and PTB. Sequential model specifications and AIC values are presented in Supplementary Table S2 for transparency. The final model adjusted for maternal age, ethnicity, infant gender, parity, migrant status, window-specific mean temperature, precipitation, wind speed, season of conception, and district fixed effects. Two-way cluster-robust standard errors by district and LMP year-month were used to address correlations among pregnancies within the same district or conception period.

The final model was specified as:

$$\text{Logit}(P(Y_{i}=1))=\beta_{0}+\beta_{1}*(\frac{PM_{2.5,i}}{10})+\sum_{k=2}^{K}\beta_{k}X_{\mathrm{ki}}+\sum_{s=1}^{3}\gamma_{s}S_{\mathrm{si}}+\sum_{d=1}^{19}\delta_{d}D_{\mathrm{di}}$$

where $Y_{i}$ indicates the preterm birth (PTB) status for pregnancy i (1 = preterm birth, 0 = term birth), and $P(Y_{i}=1)$ represents the probability of PTB. $PM_{2.5i}$ represents maternal PM_2.5_ exposure during pregnancy or a specific gestational window, $X_{\mathrm{ki}}$ represents the *k-th* covariate for pregnancy *i,* including maternal age, ethnicity, infant gender, parity, migrant status, and window-specific mean temperature, precipitation, and wind speed. $S_{\mathrm{si}}$ and $D_{\mathrm{di}}$ denote indicator variables for season of conception and residential district, respectively. $\beta_{0}$ is the intercept, representing the baseline log odds of PTB when all covariates are equal to zero. $\beta_{1}$ represents the regression coefficient for PM_2.5_ exposure, and $\beta_{k}$ represents the corresponding regression coefficient. The 95% CIs and *p* values were calculated using two-way cluster-robust standard errors by district and LMP year-month. Three season indicators and 19 district indicators were included, with one category from each variable used as the reference. Exploratory subgroup analyses based on the satellite-based whole-pregnancy PM_2.5_ exposure model were conducted by maternal age, ethnicity, infant gender, parity, and migrant status, with interaction *p* values adjusted using the Benjamini-Hochberg method.

Statistical analyses were performed using R 4.3.2. Multivariable logistic regression models were fitted using the *glm()* function from the *stats* package.

## 3 Results

### 3.1 Characteristics of study population

Of the 35,296 singleton birth records collected between January 1, 2015, and December 31, 2024, 159 records that did not meet the live-birth eligibility criterion and one record with an implausible gestational age (<140 or >350 days) were excluded. The final analysis included 35,136 singleton births, including 2,622 PTBs (7.46%) and 32,514 term births (92.54%) (Table 1).

**Table 1:** Characteristics of term and preterm births in the hospital-based cohort, 2015–2024.

Characteristics	Subgroup	Term birth (≥37 weeks)	Preterm birth (<37 weeks)	*p* value
Total, n(%)		32,514 (92.54%)	2,622 (7.46%)	
Maternal age, years		28.00 [26.00, 31.00]	29.00 [26.00, 32.00]	<0.001
Ethnic, n (%)	Han	28,764 (88.5%)	2,188 (83.4%)	<0.001
	non-Han	3,750 (11.5%)	434 (16.6%)	
Infant gender, n (%)	Female	15,825(48.7%)	1,145(43.7%)	<0.001
	Male	16,689(51.3%)	1,477(56.3%)	
Parity, n (%)	0	19,088(58.7%)	1,603(61.1%)	0.016
	≥1	13,426(41.3%)	1,019(38.9%)	
Migrant status, n (%)	Local	29,268(90.0%)	2,293(87.5%)	<0.001
	Migrant	3,246(10.0%)	329(12.5%)	
Temperature (°C)		17.69 [15.82, 19.88]	17.59 [15.09, 20.28]	0.001
Precipitation (mm)		3.25 [2.45, 3.85]	3.31 [1.91, 3.98]	0.001
Windspeed (m/s)		1.34 [1.29, 1.39]	1.33 [1.29, 1.38]	0.019
Season of conception, n (%)	Winter	8,852 (27.2%)	634 (24.2%)	<0.001
	Spring	8,165 (25.1%)	719 (27.4%)	
	Summer	7,972 (24.5%)	598 (22.8%)	
	Autumn	7,525 (23.1%)	671 (25.6%)	

Original LMP information was available for 34,389 births (97.87%) and unavailable for 747 births (2.13%). For records without original LMP information, the calculated LMP date was derived from the delivery date and the gestational age recorded in the clinical database. Compared with term births, PTB cases had a higher median maternal age (29 vs. 28 years, *p* < 0.001) and higher proportions of non-Han mothers (16.6% vs. 11.5%, *p* < 0.001), male infants (56.3% vs. 51.3%, *p* < 0.001), nulliparous women (61.1% vs. 58.7%, *p* = 0.016), and migrant women (12.5% vs. 10.0%, *p* < 0.001). Meteorological conditions and season of conception also differed between the two groups (all *p* < 0.05).

### 3.2 Spatial distribution and agreement of PM_2.5_ exposure estimates

**Figure 2:**
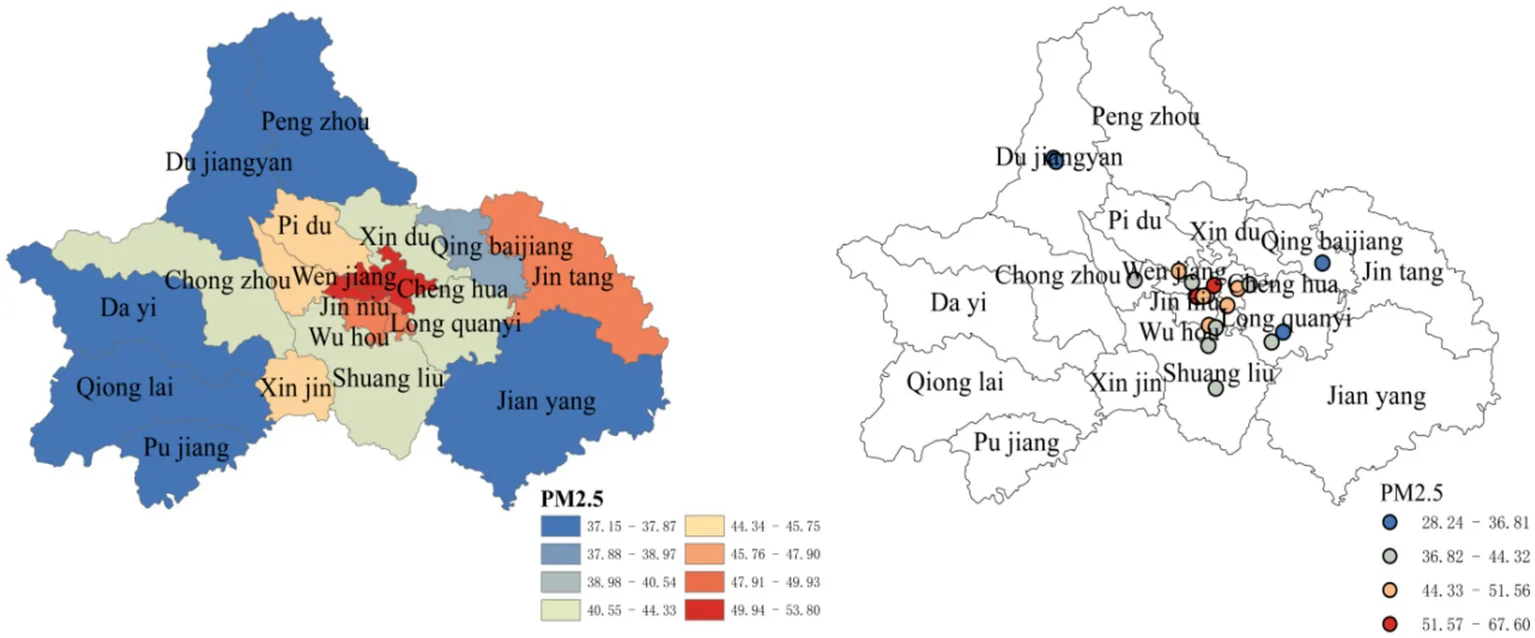
Spatial distribution of average satellite-based PM_2.5_ concentrations and ground monitoring stations in Chengdu, Southwest China.

District-level mean satellite-based PM_2.5_ concentrations ranged from 37.15 to 53.80 *ug/m^3^* (Figure 2). Concentrations were higher in central Chengdu and lower in the western and southwestern districts. The monitoring network comprised 23 stations in 11 of the 20 study districts, with site-specific means ranging from 28.24 to 67.60 *ug/m^3^*. Maternal exposure distributions across gestational windows are reported in Table 2. Monitor-based mean concentrations were higher than satellite-based estimates across these windows. For the 28,314 paired district-day observations, the Pearson correlation coefficient was 0.919, and the mean difference between satellite- and monitor-based estimates was −0.68 *ug/m^3^*. The calibration intercept and slope were −2.205 and 1.066, respectively, with Bland–Altman limits of agreement from −26.16 to 24.80 *ug/m^3^*. Further agreement measures are reported in Supplementary Table S3.

**Table 2:** Distribution of maternal PM_2.5_ exposure across gestational windows.

Exposure source	Exposure window	N	Mean ± SD, *ug/m^3^*	Median [IQR], *ug/m^3^*	Range, *ug/m^3^*
Monitor-based PM_2.5_	First trimester	35,136	52.84 ± 20.37	48.41 [36.99, 66.42]	14.97–129.08
	Second trimester	35,136	51.51 ± 20.15	47.36 [35.90, 65.07]	16.47–123.24
	Third trimester	35,136	51.43 ± 21.88	46.26 [34.13, 65.77]	10.49–142.90
	Entire pregnancy	35,136	51.91 ± 11.19	50.56 [43.55, 60.94]	21.07–83.25
	Weeks 0–27	35,136	52.13 ± 14.62	50.46 [41.02, 62.40]	19.25–93.67
	Weeks 28–31	35,065	51.33 ± 24.85	45.63 [32.56, 63.91]	11.89–151.09
	Weeks 32–33	34,907	51.43 ± 26.80	45.10 [32.17, 63.52]	9.02–173.32
	Weeks 34–35	33,934	51.38 ± 26.60	45.10 [32.26, 63.57]	8.96–171.31
Satellite-based PM_2.5_	First trimester	35,136	50.11 ± 19.76	46.81 [35.34, 62.85]	16.49–114.60
	Second trimester	35,136	48.74 ± 19.76	45.01 [32.85, 61.54]	16.12–109.81
	Third trimester	35,136	48.50 ± 21.29	43.28 [31.44, 62.02]	9.78–126.10
	Entire pregnancy	35,136	49.11 ± 11.38	47.17 [40.37, 59.03]	21.00–78.38
	Weeks 0–27	35,136	49.38 ± 14.56	48.58 [37.65, 58.09]	18.77–84.22
	Weeks 28–31	35,065	48.41 ± 23.94	42.69 [30.10, 61.25]	13.77–138.63
	Weeks 32–33	34,907	48.49 ± 25.38	42.45 [29.98, 60.29]	11.22–157.91
	Weeks 34–35	33,934	48.51 ± 25.27	42.44 [30.12, 60.46]	11.22–157.91

The distributions for weeks 28–31, 32–33, and 34–35 were calculated among pregnancies ongoing at weeks 32, 34, and 36, respectively, consistent with the corresponding landmark analyses.

### 3.3 Association between PM_2.5_ exposure and PTB

**Figure 3:**
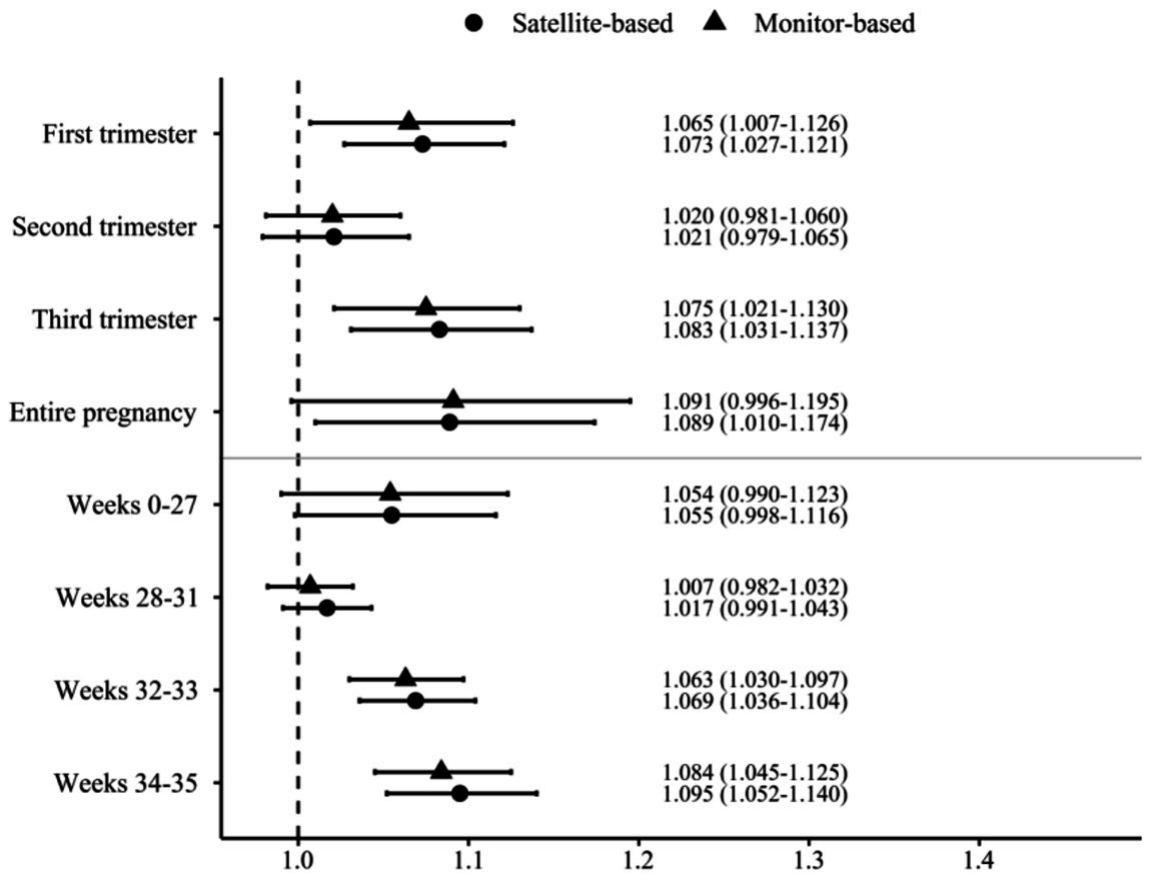
Adjusted odds ratios for preterm birth per 10 μ*g/m*^3^ increase in satellite- and monitor-based PM_2.5_ across gestational exposure windows.

Table 3 and Figure 3 present the associations between maternal PM_2.5_ exposure and PTB across conventional and fixed gestational windows. In the conventional-window analyses, monitor-based PM_2.5_ exposure was positively associated with PTB during the first trimester (OR = 1.065, 95% CI: 1.007–1.126) and third trimester (OR = 1.075, 95% CI: 1.021–1.130), but not during the second trimester or whole pregnancy. Satellite-based PM_2.5_ exposure was positively associated with PTB during the first trimester (OR = 1.073, 95% CI: 1.027–1.121), third trimester (OR = 1.083, 95% CI: 1.031–1.137), and whole pregnancy (OR = 1.089, 95% CI: 1.010–1.174). No statistically significant association was observed during the second trimester.

**Table 3:** Associations between maternal PM_2.5_ exposure and PTB across conventional and fixed gestational windows.

Exposure source	Window type	Exposure window	N	n(preterm)	Adjusted OR (95% CI)	*p*
Monitor-based PM_2.5_	Conventional windows	First trimester	35,136	2,622	1.065 (1.007–1.126)	0.029
		Second trimester	35,136	2,622	1.020 (0.981–1.060)	0.299
		Third trimester	35,136	2,622	1.075 (1.021–1.130)	0.008
		Entire pregnancy	35,136	2,622	1.091 (0.996–1.195)	0.060
	Fixed windows	Weeks 0–27	35,136	2,622	1.054 (0.990–1.123)	0.097
		Weeks 28–31	35,065	2,551	1.007 (0.982–1.032)	0.577
		Weeks 32–33	34,907	2,393	1.063 (1.030–1.097)	<0.001
		Weeks 34–35	33,934	1,420	1.084 (1.045–1.125)	<0.001
Satellite-based PM_2.5_	Conventional windows	First trimester	35,136	2,622	1.073 (1.027–1.121)	0.003
		Second trimester	35,136	2,622	1.021 (0.979–1.065)	0.319
		Third trimester	35,136	2,622	1.083 (1.031–1.137)	0.003
		Entire pregnancy	35,136	2,622	1.089 (1.010–1.174)	0.028
	Fixed windows	Weeks 0–27	35,136	2,622	1.055 (0.998–1.116)	0.058
		Weeks 28–31	35,065	2,551	1.017 (0.991–1.043)	0.195
		Weeks 32–33	34,907	2,393	1.069 (1.036–1.104)	<0.001
		Weeks 34–35	33,934	1,420	1.095 (1.052–1.140)	<0.001

In the fixed-window analyses, no statistically significant association was observed during weeks 0–27 for either monitor-based (OR = 1.054, 95% CI: 0.990–1.123) or satellite-based PM_2.5_ (OR = 1.055, 95% CI: 0.998–1.116), although both estimates were positive. No association was observed during weeks 28–31 using either monitor-based (OR = 1.007, 95% CI: 0.982–1.032) or satellite-based estimates (OR = 1.017, 95% CI: 0.991–1.043).

Among pregnancies ongoing at week 34, exposure during weeks 32–33 was associated with subsequent late PTB using both monitor-based (OR = 1.063, 95% CI: 1.030–1.097) and satellite-based estimates (OR = 1.069, 95% CI: 1.036–1.104). Among pregnancies ongoing at week 36, exposure during weeks 34–35 was associated with PTB occurring during week 36, with ORs of 1.084 (95% CI: 1.045–1.125) and 1.095 (95% CI: 1.052–1.140), respectively.

ORs were estimated per 10 *ug/m^3^* increase in PM_2.5_. Models were adjusted for maternal age, ethnicity, infant gender, parity, migrant status, window-specific temperature, precipitation, wind speed, season of conception, and district fixed effects. Standard errors were two-way clustered by district and LMP year-month. Estimates were combined across 20 gestational-age imputations using Rubin’s rules. The weeks 28–31, 32–33, and 34–35 analyses were restricted to pregnancies ongoing at weeks 32, 34, and 36, respectively. These landmark estimates represent conditional associations in different risk sets and should not be directly compared across gestational windows.

### 3.4 Associations between fixed-window PM_2.5_ exposure and PTB severity

Among the 2,622 PTB cases, 71 (2.71%) occurred at 28–31 completed weeks, 158 (6.03%) at 32–33 completed weeks, and 2,393 (91.27%) at 34–36 completed weeks. No births occurred before 28 completed weeks. Because of the small numbers in the first two categories, they were combined as earlier PTB at 28–33 weeks (*n* = 229), while births at 34–36 weeks were classified as late PTB (*n* = 2,393).

PM_2.5_ exposure during weeks 0–27 was not associated with earlier PTB using either monitor-based (OR = 0.933, 95% CI: 0.818–1.063; *p* = 0.280) or satellite-based estimates (OR = 0.939, 95% CI: 0.822–1.072; *p* = 0.331). For late PTB, positive estimates were obtained using both monitor-based (OR = 1.067, 95% CI: 1.000–1.138; *p* = 0.050) and satellite-based PM_2.5_ (OR = 1.067, 95% CI: 1.007–1.131; *p* = 0.030). Thus, the positive estimates for exposure during weeks 0–27 were confined to late PTB rather than earlier PTB (Supplementary Table S4).

### 3.5 Exploratory subgroup analyses

**Figure 4:**
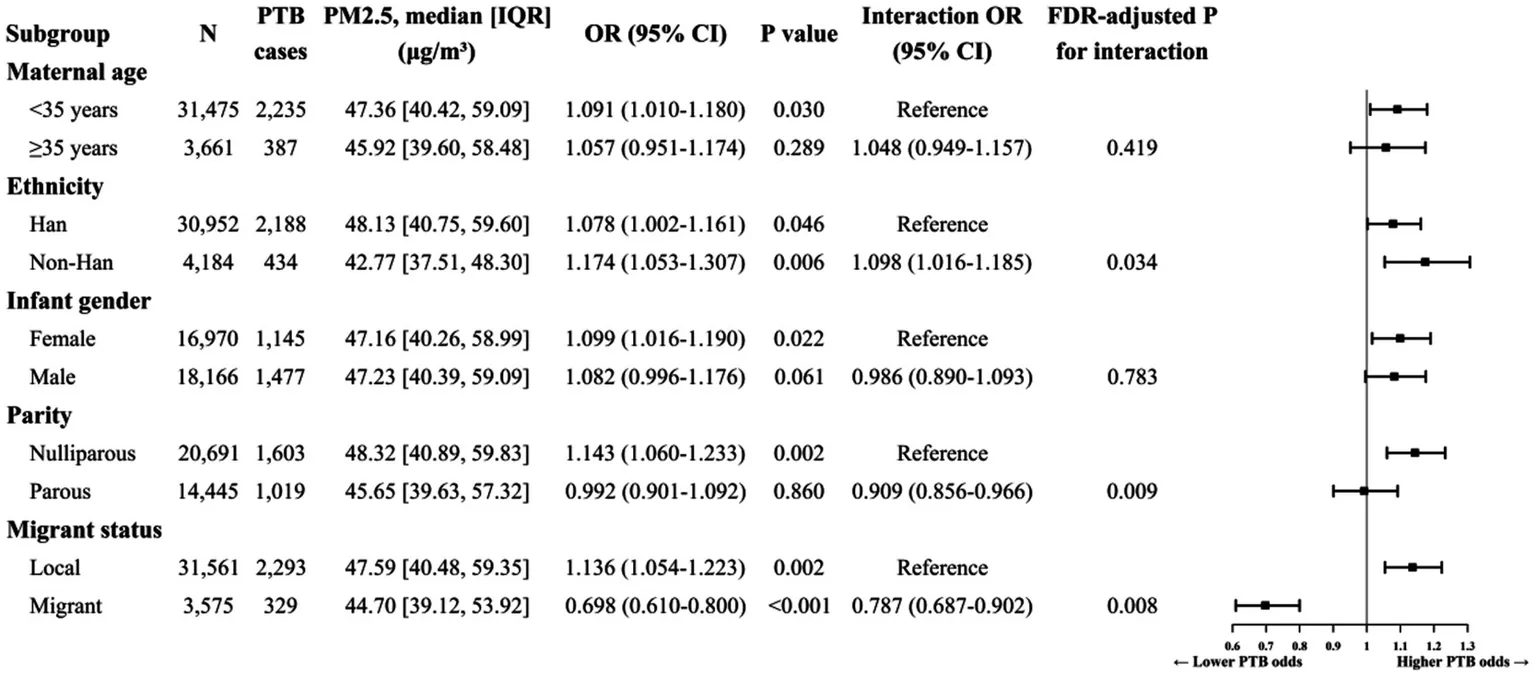
Exploratory subgroup analyses of the association between satellite-based whole-pregnancy PM_2.5_ exposure and preterm birth.

Figure 4 showed exploratory subgroup analyses of satellite-based whole-pregnancy PM_2.5_ exposure. After Benjamini-Hochberg adjustment, evidence of interaction was observed for ethnicity (interaction OR = 1.098, 95% CI: 1.016–1.185; FDR-adjusted *p* = 0.034), parity (interaction OR = 0.909, 95% CI: 0.856–0.966; FDR-adjusted *p* = 0.009), and migrant status (interaction OR = 0.787, 95% CI: 0.687–0.902; FDR-adjusted *p* = 0.008). No evidence of interaction was found for maternal age (FDR-adjusted *p* = 0.419) or infant gender (FDR-adjusted *p* = 0.783).

The association was stronger among non-Han mothers (OR = 1.174, 95% CI: 1.053–1.307) than among Han mothers (OR = 1.078, 95% CI: 1.002–1.161). A positive association was observed among nulliparous women (OR = 1.143, 95% CI: 1.060–1.233), whereas the estimate was close to the null among parous women (OR = 0.992, 95% CI: 0.901–1.092). Among local residents, the OR was 1.136 (95% CI: 1.054–1.223), whereas an inverse association was observed among migrant women (OR = 0.698, 95% CI: 0.610–0.800). The estimates were positive in both maternal-age and infant-gender strata, with no evidence of interaction for these characteristics.

## 4 Discussion

The study population experienced high PM_2.5_ exposure throughout pregnancy, with mean whole-pregnancy concentrations of 49.11 *ug/m^3^* based on satellite data and 51.91 *ug/m^3^* based on monitoring data. Positive associations were observed in the landmark analyses for PM_2.5_ exposure during weeks 32–33 and 34–35. For exposure during weeks 32–33, the ORs for late PTB at 34–36 weeks were 1.063 and 1.069 using monitoring and satellite data, respectively. For exposure during weeks 34–35, the corresponding ORs for PTB at week 36 were 1.084 and 1.095. Conventional-window analyses also showed positive associations between PM_2.5_ exposure during the first and third trimesters and PTB, whereas no statistically significant associations were observed for the fixed windows of weeks 0–27 or 28–31. In analyses by PTB severity, positive estimates were observed for late PTB but not for earlier PTB. Exploratory subgroup analyses indicated variation in the association by ethnicity, parity, and migrant status.

Previous studies have reported different PM_2.5_ exposure windows in relation to PTB (Supplementary Table S5). Li et al. (8) found stronger associations during early and mid-pregnancy and throughout pregnancy, whereas Guo et al. (9) and Zhou et al. (11) reported associations mainly during the third trimester or late pregnancy. In our study, conventional-window analyses showed positive associations between PM_2.5_ exposure during the first and third trimesters and PTB. In the fixed-window analyses, positive associations were observed during weeks 32–33 and 34–35. The results are consistent with the stronger estimates during the second and third trimesters reported by Picciotto et al. (16) and the association at week 36 identified in another California study. Averaging exposure over conventional gestational windows may obscure associations confined to shorter periods. Similarly, averaging exposure across weeks 0–27 may dilute an association specific to the first trimester. Positive associations were observed in the landmark analyses for PM_2.5_ exposure during weeks 32–33 and 34–35. These findings suggest that PM_2.5_ exposure during specific late-pregnancy periods may be associated with subsequent PTB among pregnancies that remain ongoing at the corresponding landmark points. Different biological processes may underlie the associations observed at different stages of pregnancy. Early-pregnancy exposure may affect placental development by disrupting trophoblast invasion, placental vascular remodeling, and maternal-fetal immune regulation. In late pregnancy, PM_2.5_-induced oxidative stress, placental inflammation, and endothelial dysfunction may promote the release of proinflammatory cytokines, prostaglandins, and matrix metalloproteinases, leading to cervical ripening, uterine contractions, and changes in the fetal membranes (17, 18). The associations observed during the late fixed-window analyses and for late PTB are biologically plausible and may reflect potential effects of PM_2.5_ exposure on pathways related to labor initiation.

When exposure was assigned at the district level, the effect estimates derived from satellite- and monitor-based PM_2.5_ data were similar in magnitude. In the full cohort, the ORs for whole-pregnancy exposure were 1.089 (95% CI: 1.010–1.174) and 1.091 (95% CI: 0.996–1.195), respectively, although the satellite-based estimate performed a narrower confidence interval. The two datasets were highly correlated at the district-day level (Pearson r = 0.919), with a mean satellite-minus-monitor difference of −0.68 *ug/m^3^*. However, the Bland–Altman limits of agreement ranged from −26.16 to 24.80 *ug/m^3^*, indicating differences in individual district-day estimates. The two datasets differed mainly in exposure coverage and statistical precision. ChinaHighPM_2.5_ provided continuous estimates for all 20 study districts and dates, whereas direct monitoring observations were available in only 11 districts and accounted for 35.23% of all district-day records. The remaining concentrations required spatial or temporal estimation, increasing uncertainty in exposure assignment. Xiao et al. (13) also found that gap-filled satellite data with more complete coverage produced PTB estimates comparable to those based on monitoring data but with narrower confidence intervals. Hyder et al. (12) reported that the influence of exposure-assessment methods on effect estimates may vary across birth outcomes. Among the 29,889 pregnancies with original monitoring observations, the whole-pregnancy ORs were 1.095 (95% CI: 1.056–1.136) for satellite-based data, 1.107 (95% CI: 1.057–1.159) for filled monitoring data, and 1.099 (95% CI: 1.044–1.158) for unfilled monitoring data (Supplementary Table S6). The point estimates were close and the confidence intervals overlapped substantially. The filled monitoring estimate had a narrower confidence interval than the unfilled estimate, while the satellite-based estimate had the narrowest interval. Filling monitoring gaps therefore had little influence on the magnitude of the whole-pregnancy association, whereas more complete exposure information improved statistical precision. The two datasets also yielded similar estimates in the fixed-window analyses (monitor- and satellite-based ORs: 1.054 and 1.060 for weeks 0–27; 1.007 and 1.010 for weeks 28–31; 1.063 and 1.069 for weeks 32–33; and 1.084 and 1.095 for weeks 34–35). Continuous satellite-based estimates may therefore serve as the primary exposure data source for district-level studies in areas with incomplete monitoring coverage.

Exploratory subgroup analyses identified significant interactions for ethnicity, parity, and migrant status after Benjamini-Hochberg adjusting. The association was stronger among non-Han mothers (OR = 1.174, 95% CI: 1.053–1.307) than among Han mothers (OR = 1.078, 95% CI: 1.002–1.161), with an interaction OR of 1.098 (95% CI: 1.016–1.185; FDR-adjusted *p* = 0.034). Previous studies have also reported differences in PM_2.5_-associated PTB risk across racial or ethnic groups. Such differences may reflect variation in residential environment, occupational exposure, socioeconomic conditions, and access to prenatal care rather than inherent biological susceptibility (19). The association was also stronger among nulliparous women (OR = 1.143, 95% CI: 1.060–1.233) than among parous women (OR = 0.992, 95% CI: 0.901–1.092), with an interaction OR of 0.909 (95% CI: 0.856–0.966; FDR-adjusted *p* = 0.009). A birth cohort study in northern Thailand reported a similar result, with aORs of 1.12 and 1.04 among nulliparous and parous women, respectively (20). Before a first delivery, pregnancy involves the establishment of maternal immune tolerance, trophoblast invasion, spiral artery remodeling, and placental blood flow. Oxidative stress, inflammation, and endothelial injury caused by PM_2.5_ may interfere with these processes and increase susceptibility among nulliparous women (21, 22). Among local residents, the OR was 1.136 (95% CI: 1.054–1.223), whereas an inverse estimate was observed among migrant women (OR = 0.698, 95% CI: 0.610–0.800). The interaction OR was 0.787 (95% CI: 0.687–0.902; FDR-adjusted *p* = 0.008). This result differs from that reported by Liang et al. in other Chinese cities, where the association was stronger among migrant women (23). Migrant women may change their residence, workplace, or daily activity patterns more frequently during pregnancy, increasing the difference between assigned and actual exposure and weakening the observed association. No significant interaction was found for maternal age or infant gender, although the estimates were positive in both age groups and for both female and male infants. Exploratory subgroup analyses based on whole-pregnancy PM_2.5_ exposure suggested potential heterogeneity in the association between PM_2.5_ exposure and PTB by ethnicity, parity, and migrant status. The observed differences suggest that the association between PM_2.5_ exposure and PTB may vary across population characteristics.

This study has several limitations. First, district fixed effects controlled for some area-level differences. However, residual confounding from socioeconomic, lifestyle, and clinical factors may remain. Second, adjustment for monitor-based PM_10_ and O_3_ did not materially change the PM_2.5_ estimates (Supplementary Table S7). The joint effects of multiple pollutants also require further study. Finally, clinical information on PTB subtypes was unavailable, preventing separate analyses of spontaneous, membrane rupture-related, and medically indicated PTB. Future studies should collect detailed obstetric information to examine these subtypes separately.

## 5 Conclusion

In this hospital-based cohort, maternal PM_2.5_ exposure was associated with higher odds of PTB. For exposure during weeks 32–33, the ORs for late PTB were 1.063 and 1.069 using monitor- and satellite-based data, respectively. For exposure during weeks 34–35, the corresponding ORs for PTB at week 36 were 1.084 and 1.095. Positive associations were also observed during the first and third trimesters. Analyses by PTB severity identified positive estimates for late (34–36 weeks) but not earlier (28–33 weeks) PTB. At the district level, the two exposure datasets yielded similar effect estimates, while the satellite data provided continuous coverage across all 20 districts and greater statistical precision. Exploratory analyses suggested potential heterogeneity by ethnicity, parity, and migrant status. Reducing maternal PM_2.5_ exposure throughout pregnancy may help decrease the burden of PTB.

## Data Availability

The raw data supporting the conclusions of this article will be made available by the authors, without undue reservation.

## Footnotes

1
https://zenodo.org/record/6398971

2
http://www.cnemc.cn/

3
https://cds.climate.copernicus.eu/
