# Supplementary material for: Association between ambient PM2.5 exposure during pregnancy and risk of preterm birth: a retrospective birth cohort study in Chengdu, Southwest China
Source: Front Public Health. 2026 Sep 9;14:1936547. doi: 10.3389/fpubh.2026.1936547 (PMC13597748; doi:10.3389/fpubh.2026.1936547)
Supplement: Supplementary file 1 [file Table_1.docx]

**Title Page**

**Association Between Ambient PM_2.5_ Exposure During Pregnancy and Risk of Preterm Birth: A Retrospective Birth Cohort Study in Chengdu, Southwest China**

Zhang Yi^a†*^, Lv Keyao^b†^, Yuan Yuping^a^, Li Ruifeng^a^, Li Xinbin^a^

^a^ Wuhou District People’s Hospital, Chengdu, Sichuan/Wuhou District Health Hospital for Women & Children, Chengdu, Sichuan, China.

^b^ Department of Occupational and Radiological Prevention and Control, Mianyang Center for Disease Control and Prevention, Mianyang, Sichuan, China.

^†^ These authors contributed equally to this work.

^*^ Corresponding author:

E-mail addresses: [1216520405@qq.com](mailto:gordonrozen@qq.com)

**Table S1. Sources and assignment methods of monitor-based district-day PM2.5 concentrations across the 20 study districts.**

| Source of monitor-based district-day value | District-day records | Percentage |
| --- | --- | --- |
| Mean of valid stations within the district | 28,314 | 35.23% |
| IDW using the three nearest valid stations | 44,426 | 55.28% |
| Linear temporal interpolation | 4,960 | 6.17% |
| Temporal endpoint extension | 2,660 | 3.31% |
| Total | 80,360 | 100.00% |

Note: The total represents a complete district-day dataset covering 20 districts and 4,018 days from January 1, 2014, to December 31, 2024 (20 × 4,018 = 80,360)

**Table S2. Comparison of sequential model specifications for the association between whole-pregnancy PM_2.5_ exposure and preterm birth.**

| **Exposure assessment** | **Model** | **Covariate adjustment** | **N** | **OR (95% CI)** | **P value** | **Mean AIC** |
| --- | --- | --- | --- | --- | --- | --- |
| Monitoring-station PM_2.5_ | Crude model | None | 35,136 | 1.080 (1.042-1.119) | <0.001 | 18,639.0 |
|  | Model 1 | Maternal and infant characteristics | 35,136 | 1.103 (1.063-1.143) | <0.001 | 18,492.5 |
|  | Model 2 | Model 1 + meteorological factors | 35,136 | 1.083 (1.036-1.132) | <0.001 | 18,493.3 |
|  | Model 3 | Model 2 + season of conception + district fixed effects | 35,136 | 1.091 (1.041-1.143) | <0.001 | 18,478.7 |
|  | Final model | Model 3 + two-way cluster-robust standard errors | 35,136 | 1.091 (0.996-1.195) | 0.060 | 18,478.7 |
| Satellite-based PM_2.5_ | Crude model | None | 35,136 | 1.084 (1.047-1.122) | <0.001 | 18,636.6 |
|  | Model 1 | Maternal and infant characteristics | 35,136 | 1.106 (1.067-1.146) | <0.001 | 18,489.8 |
|  | Model 2 | Model 1 + meteorological factors | 35,136 | 1.091 (1.044-1.139) | <0.001 | 18,490.3 |
|  | Model 3 | Model 2 + season of conception + district fixed effects | 35,136 | 1.089 (1.041-1.139) | <0.001 | 18,478.1 |
|  | Final model | Model 3 + two-way cluster-robust standard errors | 35,136 | 1.089(1.010-1.174) | 0.028 | 18,478.1 |

**Table S3. Correlation, calibration, and agreement between satellite- and monitor-based PM_2.5_ estimates at the district-day level in 11 monitored districts.**

| Agreement metric | Result |
| --- | --- |
| Paired district-day observations | 28,314 |
| Number of districts | 11 |
| Mean satellite-based PM_2.5_ | 43.83 *ug/m³* |
| Mean monitor-based PM_2.5_ | 44.50 *ug/m³* |
| Pearson correlation coefficient | 0.919 |
| Spearman correlation coefficient | 0.887 |
| Mean difference (satellite minus monitor) | -0.68 *ug/m³* |
| Mean absolute error | 8.48 *ug/m³* |
| Root mean square error | 13.02 *ug/m³* |
| Calibration intercept | -2.205 |
| Calibration slope | 1.066 |
| Lower Bland-Altman limit of agreement | -26.16 *ug/m³* |
| Upper Bland-Altman limit of agreement | 24.80 *ug/m³* |

**Table S4. Associations of whole-pregnancy and weeks 0-27 PM_2.5_ exposure with earlier and late preterm birth.**

| Exposure source | Exposure window | Outcome | Comparison sample, N | PTB_cases | Adjusted OR (95% CI) | P |
| --- | --- | --- | --- | --- | --- | --- |
| Monitor-based PM_2.5_ | Whole pregnancy | Earlier PTB (28-33 weeks) | 32743 | 229 | 0.909 (0.749-1.103) | 0.314 |
|  |  | Late PTB (34-36 weeks) | 34907 | 2393 | 1.110 (1.011-1.218) | 0.031 |
|  | Weeks 0-27 | Earlier PTB (28-33 weeks) | 32743 | 229 | 0.933 (0.818-1.063) | 0.28 |
|  |  | Late PTB (34-36 weeks) | 34907 | 2393 | 1.067 (1.000-1.138) | 0.05 |
| Satellite-based PM_2.5_ | Whole pregnancy | Earlier PTB (28-33 weeks) | 32743 | 229 | 0.933 (0.780-1.116) | 0.427 |
|  |  | Late PTB (34-36 weeks) | 34907 | 2393 | 1.105 (1.020-1.198) | 0.017 |
|  | Weeks 0-27 | Earlier PTB (28-33 weeks) | 32743 | 229 | 0.939 (0.822-1.072) | 0.331 |
|  |  | Late PTB (34-36 weeks) | 34907 | 2393 | 1.067 (1.007-1.131) | 0.03 |

**Table S5. Comparison of Study Designs and Findings between the Present Study and Previous Studies of Prenatal PM_2.5_ Exposure and Preterm Birth.**

| Study | Setting/N | Data | Analyses | Main findings |
| --- | --- | --- | --- | --- |
| **Li et al. (2018)** | China; 1,280,524 | SAT | T1-T3, WP, SG | PM_2.5_ was associated with PTB in all GW; associations were stronger in T1, T2, and WP than in T3. |
| **Guo et al. (2018)** | China;  426,246 | MON | T1-T3, WP, SG | Positive associations were found in all GW, with the strongest association in T3 (HR = 1.06 per 10 μg/m³). |
| **Chu et al. (2021)** | Multicentre China;  5,976 | SAT | T1-T3, WP, EM | Higher PM_2.5_ exposure during T1 was associated with an increased risk of PTB. |
| **Zhou et al. (2022)** | Chongqing; 572,116 | MON | T1-T3, WP, MP | PM_2.5_ exposure was associated with PTB during T3 (RR=1.036) and WP (RR=1.101) per 10 *ug/m*³ increase. |
| **Guo & Jiang (2025)** | China;  29 studies, 9.28 million | Multiple | Meta-analysis by GW | Significant associations were found for T2, T3, and WP, but not for T1. |
| **Xiao et al. (2018)** | Shanghai; 132,783 | SAT + MON | T1-T3, WP, multiple outcomes | Gap-filled SAT estimates produced stronger associations than unfilled SAT estimates and estimates similar to MON, but with narrower CIs. |
| **Hyder et al. (2014)** | USA;  CT and MA | SAT + MON | Multiple outcomes | Effect estimates differed for BW and SGA, whereas no significant association with PTB was found using any EA method. |
| **Present study** | Chengdu; 35,136 | SAT + MON | T1-T3, WP, FW, PS, SG | Both exposure datasets showed positive associations with PTB during T1, T3, and the late fixed windows (weeks 32-33 and 34-35), but not during T2 or weeks 28-31. Whole-pregnancy and weeks 0-27 estimates were less consistent across datasets. Positive estimates in the severity analysis were limited to late PTB, and exploratory heterogeneity was observed by ethnicity, parity, and migrant status. |

Abbreviations: SAT, satellite-based; MON, monitor-based; T1-T3, first to third trimesters; WP, whole pregnancy; FW, fixed gestational windows; PS, PTB severity analysis; SG, subgroup analysis; EM, effect modification; MP, multipollutant analysis; GW, gestational window; EA, exposure assessment; BW, birth weight; SGA, small for gestational age; CT, Connecticut; MA, Massachusetts; CI, confidence interval.

**Table S6. Sensitivity analysis comparing whole-pregnancy PM_2.5_ estimates from three exposure datasets.**

| Exposure dataset | Exposure-data processing | N | PTB cases | Adjusted OR (95% CI) | P |
| --- | --- | --- | --- | --- | --- |
| Satellite-based PM_2.5_ | Continuous district-level estimates | 29889 | 2278 | 1.095 (1.056-1.136) | <0.001 |
| Filled monitor-based PM_2.5_ | Monitoring data completed using spatial and temporal estimation | 29889 | 2278 | 1.107 (1.057-1.159) | <0.001 |
| Unfilled monitor-based PM_2.5_ | Original monitoring observations only | 29889 | 2278 | 1.099 (1.044-1.158) | 0.001 |

**Table S7. Associations of whole-pregnancy PM_2.5_, PM_10_, and O_3_ exposure with preterm birth in single- and multi-pollutant models.**

| **PM_2.5_ exposure source** | **Model** | **Pollutant estimate** | **OR (95% CI)** | **P value** |
| --- | --- | --- | --- | --- |
| Monitoring-station | PM**_2.5_** | PM**_2.5_** | 1.091 (0.996-1.195) | 0.06 |
| Monitoring-station | PM_10_ | PM_10_ | 1.042 (0.991-1.097) | 0.105 |
| Monitoring-station | O_3_ | O_3_ | 0.943 (0.836-1.063) | 0.318 |
| Monitoring-station | PM**_2.5_** + PM_10_ | PM**_2.5_** | 1.167 (0.909-1.499) | 0.211 |
|  |  | PM_10_ | 0.964 (0.844-1.102) | 0.577 |
| Monitoring-station | PM**_2.5_** + O_3_ | PM**_2.5_** | 1.087 (0.990-1.194) | 0.078 |
|  |  | O_3_ | 0.983 (0.866-1.117) | 0.787 |
| Monitoring-station | PM_10_ + O₃ | PM_10_ | 1.040 (0.987-1.097) | 0.134 |
|  |  | O_3_ | 0.985 (0.867-1.119) | 0.811 |
| Monitoring-station | PM**_2.5_** + PM_10_ + O₃ | PM**_2.5_** | 1.178 (0.914-1.519) | 0.193 |
|  |  | PM_10_ | 0.957 (0.831-1.102) | 0.522 |
|  |  | O_3_ | 0.975 (0.854-1.112) | 0.69 |
| Satellite-based | PM**_2.5_** | PM**_2.5_** | 1.089 (1.010-1.174) | 0.028 |
| Satellite-based | PM**_2.5_** + PM_10_ | PM**_2.5_** | 1.196 (0.957-1.496) | 0.11 |
|  |  | PM_10_ | 0.949 (0.826-1.090) | 0.439 |
| Satellite-based | PM**_2.5_** + O_3_ | PM**_2.5_** | 1.085 (1.004-1.173) | 0.04 |
|  |  | O_3_ | 0.981 (0.867-1.112) | 0.756 |
| Satellite-based | PM**_2.5_** + PM_10_+ O_3_ | PM**_2.5_** | 1.221 (0.969-1.539) | 0.087 |
|  |  | PM_10_ | 0.935 (0.807-1.082) | 0.347 |
|  |  | O_3_ | 0.964 (0.849-1.095) | 0.554 |

ORs and 95% CIs were estimated per 10 *ug/m³* increase in each pollutant. All models were adjusted for maternal age, ethnicity, infant gender, parity, migrant status, whole-pregnancy mean temperature, precipitation, wind speed, season of conception, and district fixed effects. Standard errors were two-way clustered by district and LMP year-month. Estimates were combined across 20 gestational-age imputations using Rubin’s rules.
